# Whole-cell one-pot biosynthesis of dodecanedioic acid from renewable linoleic acid

**DOI:** 10.1186/s40643-024-00770-8

**Published:** 2024-05-23

**Authors:** Yi-Ke Qi, Jiang Pan, Zhi-Jun Zhang, Jian-He Xu

**Affiliations:** 1 State Key Laboratory of Bioreactor Engineering, School of Biotechnology, East China University of Science and Technology, 130 Meilong Road, Shanghai, 200237 China; 2 College of Food Science and Biology, Hebei University of Science and Technology, 26 Yuxiang Street, Shijiazhuang, 050018 China

**Keywords:** Linoleic acid, Dodecanedioic acid, *Escherichia coli*, Whole-cell biosynthesis, Multi-enzymatic cascade

## Abstract

**Background:**

Dodecanedioic acid (DDA), a typical medium-chain dicarboxylic fatty acid with widespread applications, has a great synthetic value and a huge industrial market demand. Currently, a sustainable, eco-friendly and efficient process is desired for dodecanedioic acid production.

**Results:**

Herein, a multi-enzymatic cascade was designed and constructed for the production of DDA from linoleic acid based on the lipoxygenase pathway in plants. The cascade is composed of lipoxygenase, hydroperoxide lyase, aldehyde dehydrogenase, and unidentified double-bond reductase in *E. coli* for the main cascade reactions, as well as NADH oxidase for cofactor recycling. The four component enzymes involved in the cascade were co-expressed in *E. coli*, together with the endogenous double-bond reductase of *E. coli*. After optimizing the reaction conditions of the rate-limiting step, 43.8 g L^− 1^ d^− 1^ of DDA was obtained by a whole-cell one-pot process starting from renewable linoleic acid.

**Conclusions:**

Through engineering of the reaction system and co-expressing the component enzymes, a sustainable and eco-friendly DDA biosynthesis route was set up in *E. coli*, which afforded the highest space time yield for DDA production among the current artificial multi-enzymatic routes derived from the LOX-pathway, and the productivity achieved here ranks the second highest among the current research progress in DDA biosynthesis.

**Graphical Abstract:**

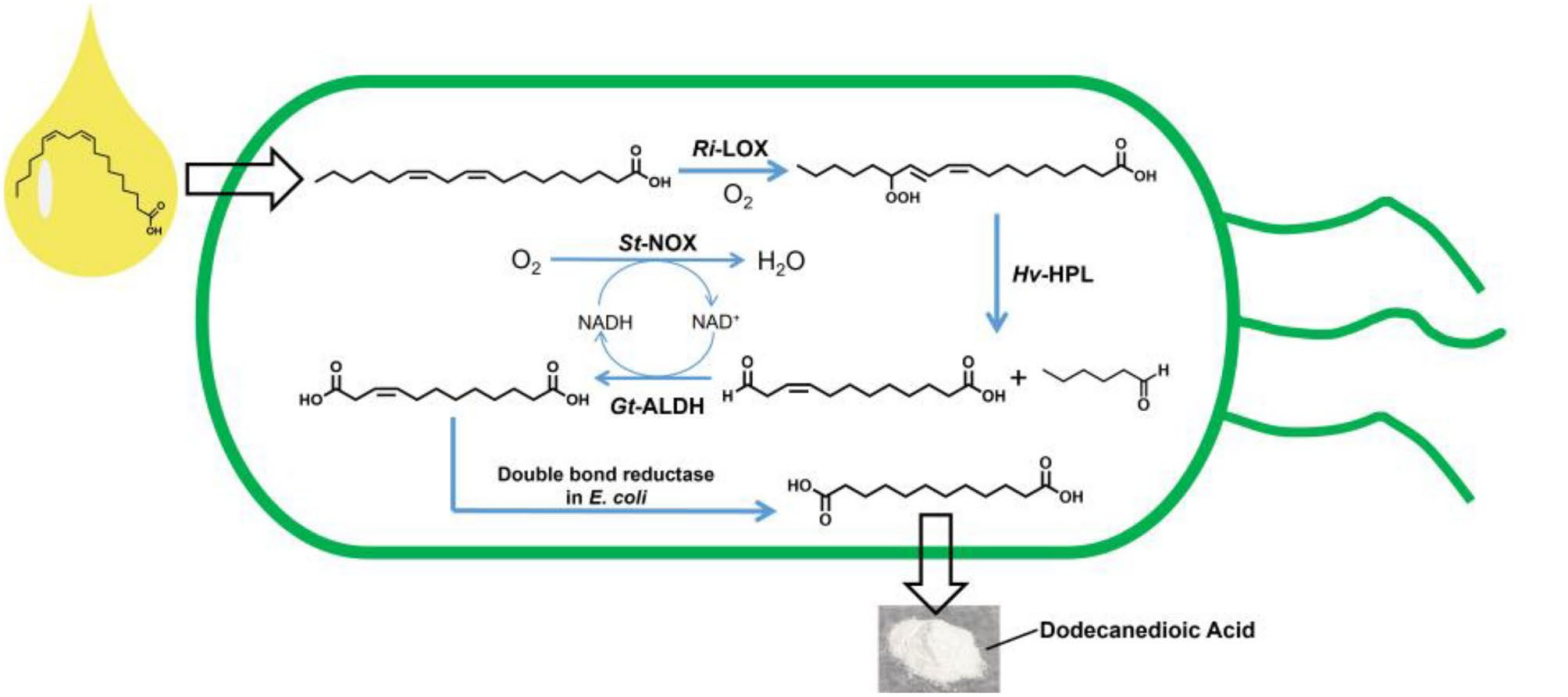

**Supplementary Information:**

The online version contains supplementary material available at 10.1186/s40643-024-00770-8.

## Introduction

The application of fatty acids and their derivatives from renewable resources as building blocks has increasingly drawn the attention of researchers from both academia and industry. Medium-chain dicarboxylic acids (C8–C12 DCAs) are of special interest, because they are the desired monomers of high-performance polyamides and polyesters (Huf et al. 2011). Dodecanedioic acid (DDA), an important medium-chain dicarboxylic fatty acid, together with aliphatic diamines, is the main building block of polyamides Nylon 6–12 and Nylon 12–12, which are important engineering plastics/fibres (Cao et al. 2017a); DDA also serves as a building block of diester compounds, which are often used for plasticiser, high grade lubricating oil and base stock (oil) (Funk et al. 2017); Furthermore, DDA is an essential functional chemical as precipitant, modifier, etc. (Werner et al. 2017). Thus, the widespread applications provide DDA a significant synthetic value.

Traditional chemical processes for the synthesis of mid-chain dicarboxylic acids suffer from harsh reaction conditions, including high temperatures and pressures. These processes usually require the use of strong acids (such as H_2_SO_4_ and HNO_3_) and toxic oxidants (such as ozone), which can cause serious safety problems and environmental pollution (Gu et al. 2024). For example, sebacic acid (C10) is produced from castor oil by energy-consuming alkaline thermal pyrolysis with relatively poor yields (Ogunniyi et al. 2006). The production of azelaic acid (C9) is based on the ozonolysis of oleic acid (Otte et al. 2014). The current industrial chemical processes for the manufacture of DDA, such as cyclohexanone oxidation and butadiene-way, give low yields and cause environmental pollution due to the use of strong acids, heavy-metal catalysts, organic solvents, and toxic agents such as hydrogen peroxide (Chen 2010). It’s worth noting that an explosion accident was caused during the industrial production of DDA from butadiene (Chen 2010). As for the fermentative DDA production, difficulties in the extraction and purification of product limited its efficiency (Chen 2010; Lee et al. 2018). On that account, safer, more environmentally benign and efficient processes would be preferable.

**Scheme 1:**
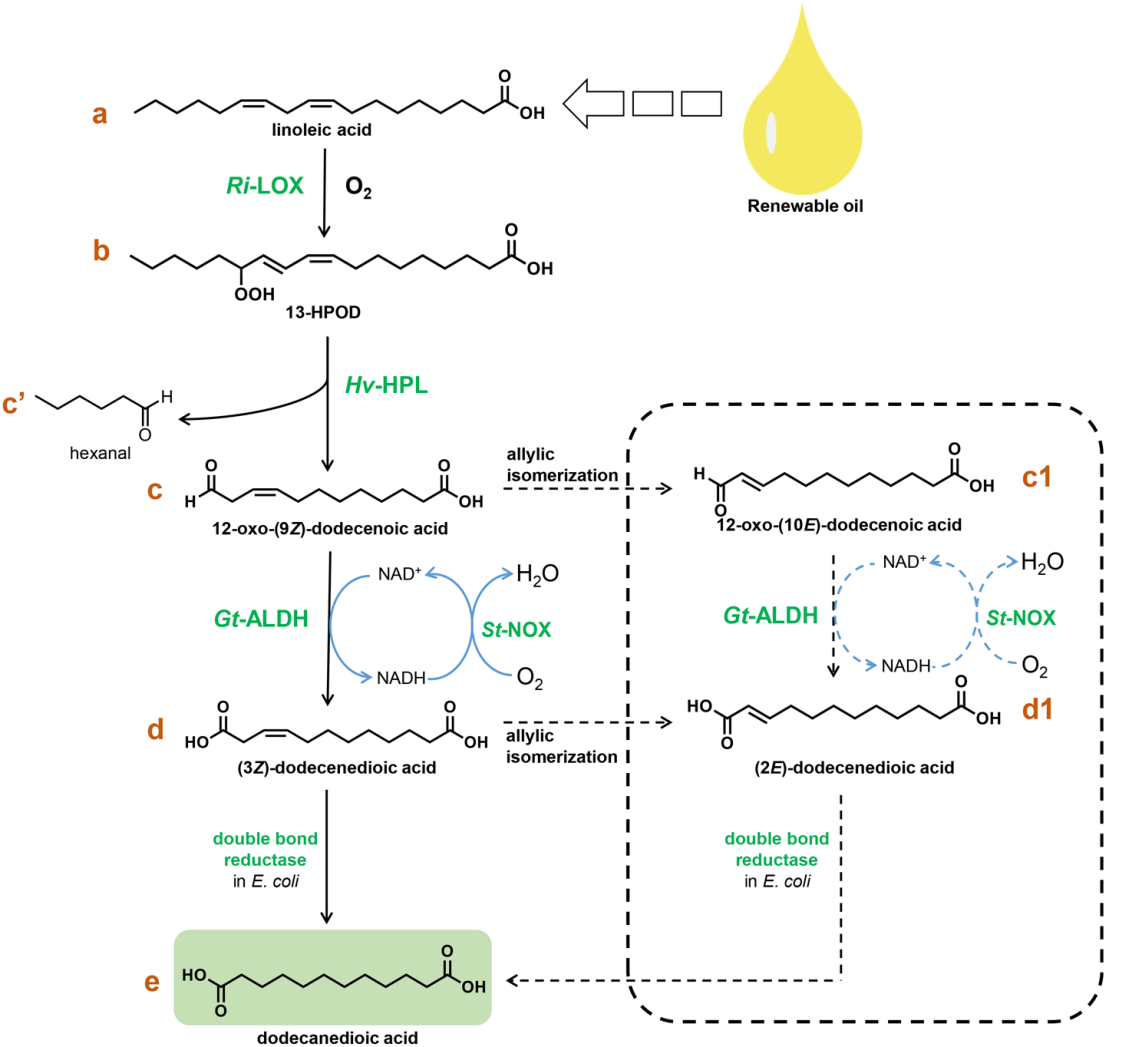
Synthesis of dodecanedioic acid from renewable linoleic acid by the cascade route proposed in this study (possible branch routes existing in the cascade reactions are shown in the dashed box)

With the development of genetic and protein engineering techniques, enzymatic processes, occurring under mild conditions and excelling by intrinsic selectivity, are particularly attractive for synthetic applications. Fascinated by the synthetic possibilities offered by various enzymes, we became focused on constructing a multi-enzymatic cascade route for the sustainable production of DDA. Based on the bio-synthesis of green leaf volatiles (GLVs) by the lipoxygenase metabolic pathway (Additional file 1: Scheme S1) in plants (Feussner et al. 2002; Matsui 2006), a multi-enzyme cascade, consisting of 13-lipoxygenase (LOX), 13-hydroperoxide lyase (HPL), aldehyde dehydrogenase (ALDH), endogenous double-bond reductase in *E. coli* and NADH oxidase (NOX), was implemented for the biosynthesis of DDA from renewable linoleic acid (LA, **a**) (Scheme 1).

LOX is a non-heme iron dioxygenase that catalyzes the insertion of oxygen into polyunsaturated fatty acids containing one or more 1,4-(*Z*,*Z*)-pentadiene motif(s) (Qi et al. 2020), such as linoleic acid. Mechanistically, the reaction occurs by hydrogen abstraction at the double allylic position C-11, followed by radical rearrangement to either position C-9 or C-13 (Ivanov et al. 2010). LOXs are therefore practically classified into 9- and 13-LOXs according to the regioselectivity of the oxygen insertion. In our previous work (Qi et al. 2020, 2021), a 13-LOX from *Rivularia* sp. PCC 7116 (*Ri*-LOX) was identified and engineered, with the highest activity (209 U/mg protein) toward linoleic acid. Therefore, the highly active *Ri*-LOX was applied to the first step of the cascade reactions. HPL is a heme iron enzyme, which belongs to the CYP74 family of the P450 macrofamily. The classification of HPLs is also based on the regioselectivity: 13-HPLs, which are only active on 13-hydroperoxide, such as 13-hydroperoxy-9,11-(*Z*,*E*)-octadecadienoic acid [13-HPOD, **b**]; While 9/13-HPLs are active on both 9- and 13-hydroperoxide, such as 9/13-HPOD (Mu et al. 2012). Herein, a 13-HPL from *Hordeum vulgare* (*Hv*-HPL) was employed for the second step in the cascade for the C–C bond cleavage of 13-HPOD produced by 13-LOX (Koeduka et al. 2003). The oxidization of the aldehyde can be accomplished by an endogenous *E. coli* oxidoreductase (Otte et al. 2014). In consideration of the reaction efficiency, an additional ALDH from *Geobacillus thermodenitrificans* (*Gt*-ALDH) was recruited for this step (Li et al. 2010). The hydrogenation reduction of the DDA precursor, which is the last step of the cascade route, was accomplished by an unidentified double bond reductase in *E. coli*, as reported previously (Kim et al. 2020). In addition, a water-forming NOX was coupled for the cofactor recycling (Sha et al. 2018).

Based on the lipoxygenase pathway, several cascade systems have been established with LOX and HPL (Buchhaupt et al. 2012; Otte et al. 2014; Coenen et al. 2022, 2023). Coenen reported a one-pot enzymatic cascade for synthesis of 12-oxo-9(*Z*)-dodecenoic acid (**c**, the precursor of DDA) starting from safflower oil (Coenen et al. 2022). The multi-enzyme system included *P. fluorescens* lipase, *Glycine max* LOX and N-terminally truncated *Carica papaya* HPL, affording a space time yield (STY) of 486.6 mg·L^–1^ d^–1^ of **c** (43% conversion of 0.67 mM linoleic acid equivalent); And by coupling only the LOX and HPL reactions, 486.0 mg·L^–1^ d^–1^ of **c** (62% conversion of 0.5 mM linoleic acid) was obtained starting from linoleic acid. Furthermore, a three-enzyme cascade comprising LOX, HPL and ω-TA was also constructed to synthesize 12-amino-9(*Z*)-dodecenoic acid with an STY of 153.6 mg·L^–1^ d^–1^ (Coenen et al. 2023). In this work, a whole-cell multi-enzymatic cascade system derived from the LOX-pathway was designed and built to produce DDA with the highest productivity among the reported LOX-pathway derived cascade routes.

Since the metabolic formation of DDA from *n*-dodecane by a mutant of *Candida tropicalis* was performed by Yi (Yi et al. 1982), a number of researches on DDA biosynthesis have been carried out (Table 1), which were based on the terminal oxidation of *n*-dodecane, dodecanoic acid or 12-hydroxy dodecanoic acid by CYPs and/or other oxidases, such as alcohol dehydrogenase and aldehyde dehydrogenase. The productivity of these biosynthetic processes ranged from 7.6 × 10^− 6^ to 44.9 g·L^–1^·d^–1^, among which only one record was above 40 g·L^–1^·d^–1^. The reasons are mainly due to the common challenges of CYPs such as poor reactivity, poor stability, and the requirement of complex cofactor-dependent electron transfer partners. The new synthetic route of DDA in this study is free of those challenges, and starts from the renewable linoleic acid for the first time. The STY achieved here ranks the second highest among the current processes for DDA bioproduction.

## Methods

### Chemicals and materials

All chemicals and reagents were purchased from authentic suppliers at reagent grade or higher purity, and were used directly without further purification. Bacterial strains [*E. coli* BL21(DE3) and *E. coli* JM109] and plasmids were obtained from our laboratory. Tryptone and yeast extract were obtained from Oxoid (Hampshire, UK). Genes were synthesized by Nanjing GenScript Biotech Co. DNA polymerases, restriction enzymes, and T4 DNA ligase were from TaKaRa (Dalian, China). DNA ligase of HiFi DNA Assembly was from New England BioLabs (England). The 5 mL HisTrap FF column was supplied by GE Healthcare.

### Gene cloning, protein expression, and purification

The genes encoding *Ri*-LOX and *Hv*-HPL were ligated into pET-21a-(+), pMal-c2X, respectively, and the genes encoding *Gt*-ALDH and *St*-NOX were ligated into pET-28a-(+), respectively, and then transformed into *E. coli* competent cells for over-expression. Positive transformants were grown at 37 °C to an optical density of 0.6 to 0.8 at 600 nm in Luria broth medium containing 100 µg/mL ampicillin (for pET-21a and pMal-c2X) or 50 µg/mL kanamycin (for pET-28a). Protein production was induced by adding isopropyl-β-D-thiogalactopyranoside (IPTG) to a final concentration of 0.2 mM (for *Hv*-HPL production, the supplementation of 0.2 mM δ-aminolevulinic acid was required), and the cells were cultured for a further 22 h at 16 °C. Cells were harvested by centrifugation (5000 × g) at 4 °C for 15 min. For protein purification, the harvested cells were resuspended in ice-chilled buffer A (25 mM Tris-HCl, 300 mM NaCl, 10 mM imidazole, pH 8.0), and then disrupted by ultrasonication. After centrifugation at 12,000 × g and 4 °C for 40 min, the supernatant was loaded onto a His-Trap Ni-nitrilotriacetic acid FF column (5 mL; GE Healthcare Co.) pre-equilibrated with buffer A. The target protein was eluted using an increasing gradient of imidazole from 10 to 200 mM at a flow rate of 5 mL/min and detected by SDS-PAGE. The fraction containing the purified protein was collected, and concentrated by ultrafiltration. After measuring the protein concentration, the freshly purified enzymes were stored at − 80 °C for further measurements.

The co-expression systems were constructed based on the MBP-*Hv*HPL expression system in *E. coli* (pMal-c2X-MBP-*Hv*HPL) for the recombinant expression of *Ri*-LOX, *Gt*-ALDH and *St*-NOX, yielding two co-expression systems: p-HRGS-1 (single promoter) and p-HRGS-2 (double promoters). The primers used for the co-expression systems construction are listed in Additional file 1: Table S1.

### Enzyme assay

The activity of *Ri*-LOX was assayed at 30 °C and pH 8.5 by monitoring the increase in absorbance at 234 nm. An extinction coefficient of 25,000 M^− 1^ cm^− 1^ was used to calculate the activity of enzyme with regard to the production of 13-HPOD (Gibian et al. 1987). The details of LOX activity assay were described previously (Qi et al. 2020). The lag periods of *Ri*-LOX-catalyzed dioxygenation reactions were assayed at 30 °C and pH 8.5 with a substrate loading of 0.5 mM and three low enzyme dosages (0.1 µg mL^− 1^, 0.05 µg mL^− 1^ and 0.025 µg mL^− 1^), respectively, by monitoring the increase in absorbance at 234 nm.

As a typical assay (1 mL), HPL activity was determined by coupling with LOX-reaction in 100 mM KPB, pH 8.0, with 0.1 mM linoleic acid dissolved in methanol (final concentration of methanol: 2%), and the decrease in absorbance at 234 nm due to the cleavage of 13-HPOD was measured.

For ALDH activity assay, the standard reaction mixture was composed of 100 mM KPB (pH 8.0), 1 mM NAD^+^, 1 mM acetaldehyde and an appropriate amount of enzyme. The mixture without the substrate was used as the blank. The assay was carried out at 30 ℃ for 1 min. The change in absorbance at 340 nm caused by the formation of NADH due to the oxidization of substrate by ALDH was monitored (extinction coefficient, ε = 6.22 × 10^3^ M^− 1^ cm^− 1^) using a UV-visible spectrophotometer. One unit of activity was defined as the amount of enzyme that catalyzed the formation of 1.0 µmol of NADH per minute.

### Enzymatic cascade reactions

The LOX-HPL cascade reaction was carried out on a 10-mL scale consisting of 20 mM linoleic acid, 0.5 U mL^− 1^ of purified *Ri*-LOX_MB_, 0.3 U mL^− 1^ of purified MBP-*Hv*HPL, 2 M of KCl and potassium phosphate buffer (KPB; 100 mM, pH 8.0). The whole-cell one-pot cascade reactions for DDA synthesis were performed on both 10-mL and 100-mL scales in KPB (100 mM, pH 8.0) containing 2 M of KCl, with a reaction setup consisted of linoleic acid (20, 50 or 100 mM), *E. coli* wet cells co-expressing *Ri*-LOX_MB_, MBP-*Hv*HPL, *Gt*-ALDH, and *St*-NOX (30 or 50 g/L), and supplied with sufficient O_2_. The reaction was started by the addition of cells/enzymes and quenched with the addition of H_2_SO_4_ (1 M).

### Analytical methods

The resulting product DDA was extracted with methyl *tert*-butyl ether (MTBE) and dissolved in methanol after complete volatilization of MTBE. Analysis of the product was performed by high-performance liquid chromatography (HPLC) with a UV detector at 210 nm and a reverse phase Nucleosil C18 column (Hypersil ODS2, 4.6 × 250 mm, 5 μm), and the column was eluted with a solvent system of methanol/water/phosphoric acid (95/5/0.1, by volume) at a flow rate of 0.4 mL min^− 1^ at 30 °C. The retention time of DDA was 7.86 min. ^1^H-NMR and ^13^C-NMR measurements were conducted on a Bruker Avance 600 MHz spectrometer.

## Results and discussion

### Design of a multi‑enzymatic cascade for converting linoleic acid to dodecanedioic acid

GLVs including short carbon chain aldehydes, alcohols, and esters are important contributors to the characteristic flavors of fruits, vegetables, and green leaves. They are produced through the lipoxygenase pathway of higher plants (Vincenti et al. 2019). Lipoxygenase pathway is composed of the dioxygenation by LOX and the C–C bond cleavage by HPL. The combination of LOX and HPL is an established enzyme system in the flavor and fragrance industry to produce GLVs (Koeduka 2018).

There are two reaction routes in the LOX pathway with LA as the substrate (Additional file 1: Scheme S1). 9-LOXs produce 9-HPOD, and 9-HPLs act on 9-hydroperoxides to form C9 aldehydes; 13-LOXs produce 13-HPOD, and 13-HPLs act on 13-hydroperoxides to form C6 aldehydes and 12-oxoacid. Inspired by the 13-LOX/HPL pathway, the cascade route (Scheme 1) was designed by adding ALDH and double bond reductase to the original pathway for DDA (**e**) production, and NOX for recycling the cofactor. It’s worth noting that the 9(*Z*)- and 3(*Z*)-double bonds in 12-oxo-9(*Z*)-dodecenoic acid (**c**) and 3(*Z*)-dodecenedioic acid (**d**) are unstable and can spontaneously isomerize to 10(*E*)- and 2(*E*)-isomers, respectively, by the allylic isomerization for forming the thermodynamically preferential conjugated oxoenes (Grechkin et al. 2002; Gargouri et al. 2004). Therefore, there are possible branch routes existing in the cascade reactions as shown in the dashed box of Scheme 1.

### Recruiting enzymes for the designed cascade reaction

*Ri*-LOX, *Hv*-HPL and *Gt*-ALDH were selected for the cascade to convert LA (**a**) into dicarboxylic acid **d** (1). The consistent and suitable pH of cascade enzymes is crucial for a one-pot system. Most HPLs characterized thus far have optimal pH values in a weakly acidic region ranging from 5.0 to 6.5, which suggests that the carboxylic acid form of the substrate is a prerequisite for HPL action (Koeduka et al. 2003). However, the optimum pH for LOX-catalyzed dioxygenation is generally alkaline, such as 8.5 for *Ri*-LOX. Thus, the rare alkaline-preferred 13-HPL, *Hv*-HPL with an optimal pH of 8.0 (Koeduka et al. 2003), was pitched on for the cascade. Alkaline environment is also acceptable for the broad-spectrum *Gt*-ALDH, which could catalyze the oxidation of fatty aldehydes efficiently (Li et al. 2010). For NAD^+^ regeneration, a water-forming NOX with an optimal pH of alkalescence from *Streptococcus thermophilus* (*St*-NOX) (Sha et al. 2018), was coupled with the third step of the cascade, ensuring the rapid oxidation of NADH to NAD^+^ (18.1 U mg^–1^) and producing innocuous water as the sole byproduct.

*Ri*-LOX, *Gt*-ALDH and *St*-NOX were well expressed in *E. coli* BL21(DE3) cells (Additional file 1: Fig. S1). The gene encoding *Hv*-HPL was initially ligated into vector pQE30 and subsequently transformed into *E. coli*, however, there was no active enzyme expressed, even after optimization of expression conditions (Additional file 1: Fig. S2B). Then vector pQE30 was replaced with pET-21a(+), resulting in enzyme expression of insoluble form (Additional file 1: Fig. S2A). For high-level functional expression of soluble HPL, the maltose-binding protein (MBP) was fused with *Hv*-HPL through the vector pMal-c2X in *E. coli* JM109 cells. With the fusion expression of MBP, high-level soluble *Hv*-HPL was expressed in the form of MBP-*Hv*HPL (Additional file 1: Fig. S2C), and used for further reactions.

### Identification of rate-limiting step

The LOX/HPL/ALDH-NOX cascade was tentatively performed on a 1 mL-scale in vitro at pH 8.0 and 30 ℃. The one-pot reactions were carried out with LA loadings of 10, 15 and 20 mM, respectively. After 1 h of reaction, LA was completely transformed and dicarboxylic acid **d** was produced as envisioned (Additional file 1: Fig. S3), demonstrating the general feasibility of the four-enzyme cascade. However, much more compound **b** was observed in the reaction mixture than compound **d** as detected by LC-MS, and no compound **c** was detected (Additional file 1: Fig. S3), indicating that the poor catalytic efficiency of MBP-*Hv*HPL (or at least under the current reaction conditions) led to the significant accumulation of compound **b**. Hence, the C–C bond cleavage of compound **b** catalyzed by MBP-*Hv*HPL was presumed as the rate-limiting step of the cascade route, which was expected to be further optimized.

### Optimization of the hydroperoxide cleavage reaction

**Fig. 1:**
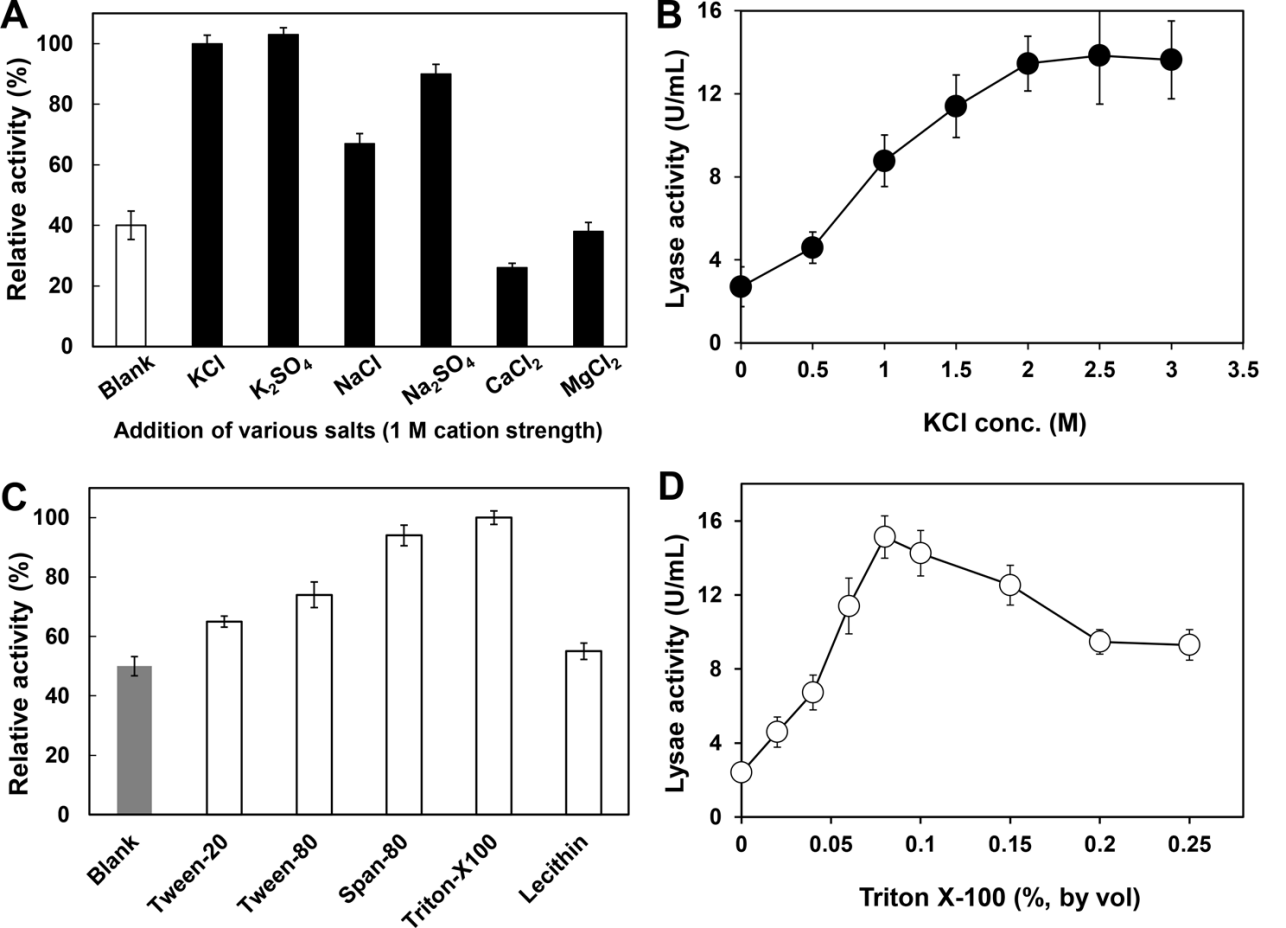
Effect of various salts (**A**) and different concentrations of KCl (**B**) on the activity of MBP-*Hv*HPL; Effect of various detergents (**C**) and different concentrations of Triton X-100 (**D**) on the activity of MBP-*Hv*HPL

To relieve the major limitation caused by the low efficiency of the cleavage reaction, the HPL-catalyzed hydroperoxide cleavage reaction was then optimized. The variety and concentration of metal ions and detergents in the reaction mixture are critical factors affecting the activity of HPLs (Noordermeer et al. 2001; Koeduka et al. 2003). Therefore, the effects of these two factors on the catalytic efficiency of MBP-*Hv*HPL were investigated and optimized. Firstly, the effect of different salts was examined according to the study of Koeduka (Koeduka et al. 2003). As shown in Fig. 1A, 6 kinds of salts with the same cation strength (1 M) were added to the reaction system and evaluated, respectively. Different from divalent cations, which are known to associate with the carboxylate anion of fatty acids and mask the carboxylate anion needed for catalysis by HPL, monovalent cations have a significant positive effect on the activity of MBP-*Hv*HPL. K^+^ is superior to Na^+^ for increasing the activity of MBP-*Hv*HPL. Taking the anion into consideration, the effect of K_2_SO_4_ was comparable to that of KCl, however, K_2_SO_4_ was eliminated due to its relatively low solubility (0.75 M, 30 ℃) compared to KCl (4.99 M, 30 ℃). The failure of increasing the HPL activity by divalent cations suggested that the terminal carboxylate anion was important for the alkaline-preferred *Hv*HPL to recognize its substrate. Furthermore, the lyase activity of MBP-*Hv*HPL was estimated in the presence of various concentrations of KCl (Fig. 1B). The results indicated that the activity of MBP-*Hv*HPL increased as the concentration of KCl in the reaction buffer increased, and the activity of MBP-*Hv*HPL tended to be steady with KCl concentration higher than 2 M (about 5-fold of blank).

According to previous research (Koeduka et al. 2003), the *V*_max_ of *Hv-*HPL increased approximately three times after the addition of 1.5 M potassium chloride, whereas the *K*_m_ value essentially was not affected, which suggests that the monovalent cation exerted the effect on the enzyme itself. In brief, the monovalent cation affected the nature of *Hv-*HPL. Similarly, the activities of some other enzymes, such as *Aspergillus niger* glucose oxidase, are also quite sensitive to such an effect, and its conformation changes in response to NaCl up to 2 M (Ahmad et al. 2001). It can be expected that monovalent cations enhance the activity of *Hv-*HPL through the global effect on the conformation.

The effect of detergents on the activity of MBP-*Hv*HPL was also investigated. Activity improvement of 1.1 ∼ 2 fold was observed in the presence of various detergents, among which Triton X-100 showed the best performance (Fig. 1C). Then, the activity was determined with various concentrations of Triton X-100 (Fig. 1D), and the highest activity (about 6-fold of blank) was achieved with a Triton X-100 concentration of 0.08% (v/v). Here, by optimizing the conditions of the hydroperoxide cleavage reaction, 2 M KCl and 0.08% (v/v) Triton X-100 were determined to be the optimal supplements of the reaction system.

### Coordination of the LOX-HPL cascade system

By optimizing the hydroperoxide cleavage reaction, the vital conditions for the conversion of 13*S*-HPOD were identified. To coordinate the LOX-HPL cascade system, which plays a vital role in the whole cascade route, the effects of KCl and Triton X-100 on the activity of *Ri*-LOX were investigated.

**Fig. 2:**
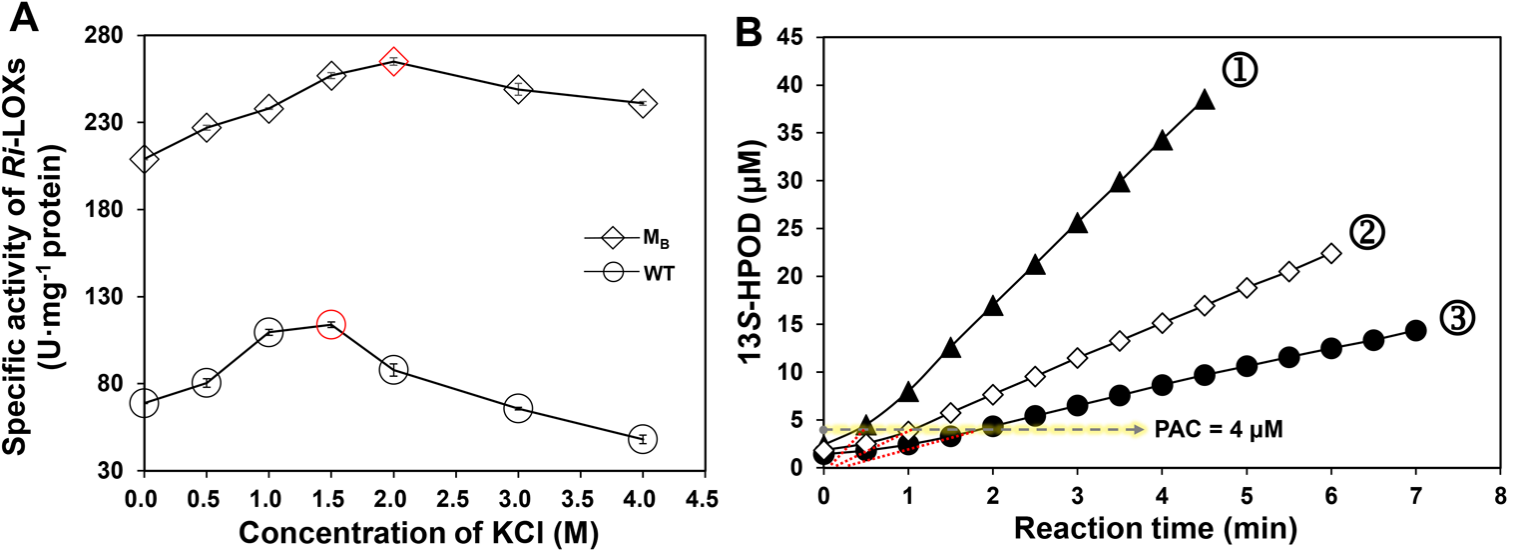
**A**: Effect of concentrations of KCl on the activity of *Ri*-LOX_WT_ and *Ri*-LOX_MB_. **B**: Measurement of the PAC in the oxygenation of linoleic acid by *Ri*-LOX_WT_ [Reaction conditions: 0.5 mM LA, purified LOX: ①: 0.1 µg/mL; ②: 0.05 µg/mL; ③: 0.025 µg/mL, KPB (100 mM, pH 8.5), 2% (v/v) methanol, 30 ℃]

The activities of *Ri*-LOX_WT_ and *Ri*-LOX_MB_ [A324G/S392G, the best mutant obtained by the oxygen channel engineering of *Ri*-LOX (Qi et al. 2021)] with different KCl concentrations in the reaction systems were determined under the standard activity assay conditions. As displayed in Fig. 2A, the activities of both *Ri*-LOX_WT_ and *Ri*-LOX_MB_ profiled as a bell-shaped curve with the increasing of KCl concentration. The activity of *Ri*-LOX_MB_ peaked at 2 M KCl with 265 U/mg. The enhancement of *Ri*-LOX activity under the high concentration of salt might result from a shift of the p*K*_a_ value of LA, and/or the global effect on the conformation modulated by the increasing ion strength in the buffer, thereby facilitating the enzymatic dioxygenation. However, when it came to the effect of detergent, the *Ri*-LOX was inactivated by Triton X-100, indicating that the detergent cannot be adopted even though its positive effect on the HPL activity.

In the LOX-HPL cascade system, LOX and HPL compete for 13-HPOD (**b**) because of the lag period of LOX (Otte et al. 2014). Oxygenation of unsaturated fatty acids by LOX occurs with a kinetic lag period at the beginning of the reaction, and the addition of an appropriate amount of product hydroperoxide (such as 13-HPOD) can abolish the lag period by activating the LOX involved (Smith et al. 1972). The amount of 13-HPOD required for abolishing the lag period is product-activating concentration (PAC). The lag period of *Ri*-LOX, which was too short to be observed in our previous work (Qi et al. 2020), was then investigated, and the PAC was measured under the condition of low LOX dosages (0.025 ∼ 0.1 mg/L). As shown in Fig. 2B, the lag periods of the dioxygenation reactions were relatively short and became shorter with the increasing of enzyme dosage. The results showed that the PAC of *Ri*-LOX was only 4 µM, indicating that the LOX was totally activated as 13-HPOD accumulated to 4 µM in the reaction mixture. According to further calculation, the LOX activity of greater than only 0.004 U/mL was enough to reduce the lag period to less than 1 min. Thus, considering the actual activity of *Ri*-LOX in the reaction system, which is much higher than 0.004 U/mL, the lag period of *Ri*-LOX could be negligible.

**Fig. 3:**
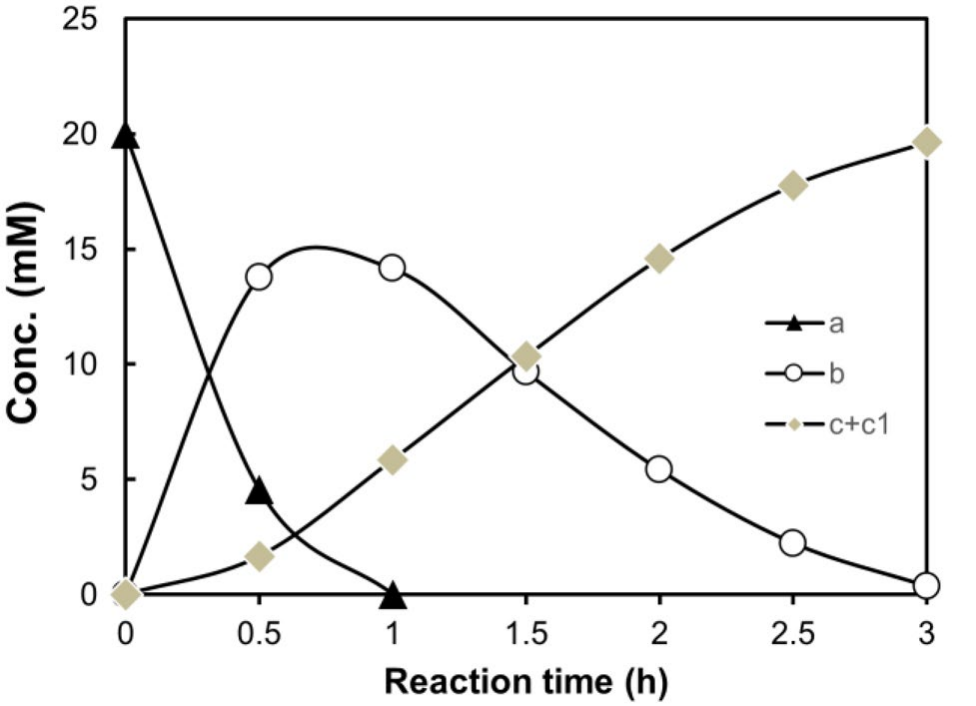
Time course of the LOX-HPL cascade reaction for the transformation of linoleic acid (**a**) into 12-oxo-(9*Z*)-dodecenoic acid (**c**). Reaction conditions (10 mL): 20 mM linoleic acid, 0.5 U mL^–1^ of purified *Ri*-LOX_MB_, 0.3 U mL^–1^ of purified MBP-*Hv*HPL, 2 M of KCl, potassium phosphate buffer (KPB; 100 mM, pH 8.0), supplied with sufficient O_2_

Based on the optimized cleavage reaction, the coordinated LOX-HPL cascade showcased the feasibility of total conversion of **a** to **c** (1), giving a decent molar yield of 98% (Fig. 3).

### Whole-cell one-pot biosynthesis of dodecanedioic acid (e)

**Fig. 4:**
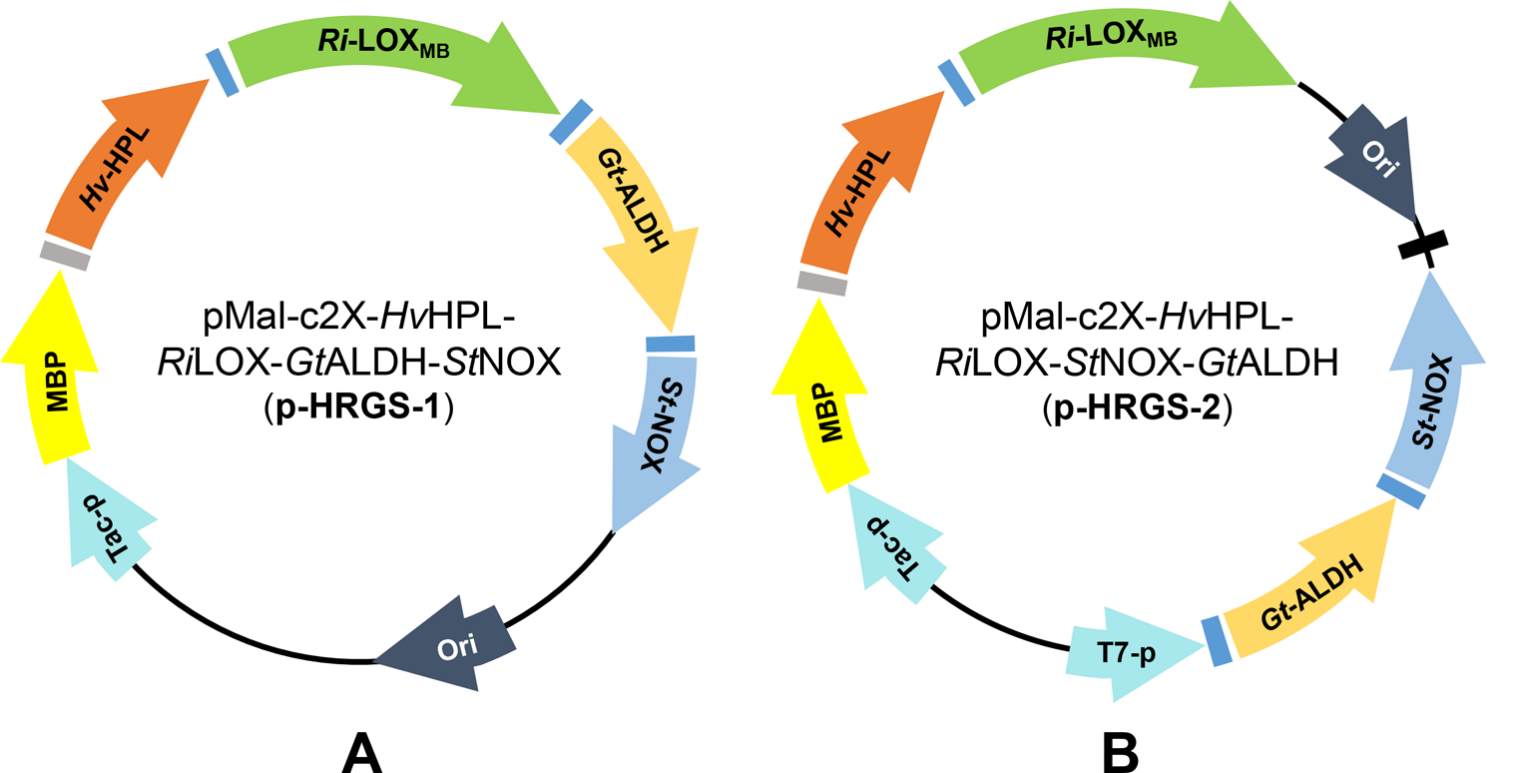
Two co-expression systems of the four enzymes: p-HRGS-1 system with the single original Tac-promoter (**A**); p-HRGS-2 system with two promoters (Tac and T7) (**B**)

For multi-enzyme cascade, considering the addition of various enzymes and the stability of the reaction system, a simpler and more stable single-cell catalyst, in which all enzymes involved in the cascade are co-expressed, tends to be pursued. Therefore, we attempted to construct a co-expression system of these component enzymes. Considering the soluble expression of *Hv*-HPL requires the fusion of MBP, the MBP-*Hv*HPL expression system in *E. coli* (pMal-c2X-MBP-*Hv*HPL) described previously was used as the chassis for the recombinant expression of *Ri*-LOX_MB_, *Gt*-ALDH and *St*-NOX, yielding a co-expression system: pMal-c2X-MBP-*Hv*HPL-*Ri*LOX_MB_-*Gt*ALDH-*St*NOX (abbreviated as p-HRGS-1, Fig. 4A). However, *Gt*-ALDH and *St*-NOX were not well expressed by the p-HRGS-1 system in both *E. coli* JM 109 and *E. coli* BL21(DE3) cells due to the limitation of single promoter (Additional file 1: Fig. S4). Then, another co-expression system was constructed by reversely introducing a new T7-promoter to the vector pMal-c2X for the expression of *Gt*-ALDH and *St*-NOX, while the expression of MBP-*Hv*HPL and *Ri*-LOX was under the control of the original Tac-promoter (abbreviated as p-HRGS-2, Fig. 4B). To our delight, four enzymes were well expressed under the control of two separate promoters by the p-HRGS-2 system in *E. coli* BL21(DE3) (Additional file 1: Fig. S4).

**Fig. 5:**
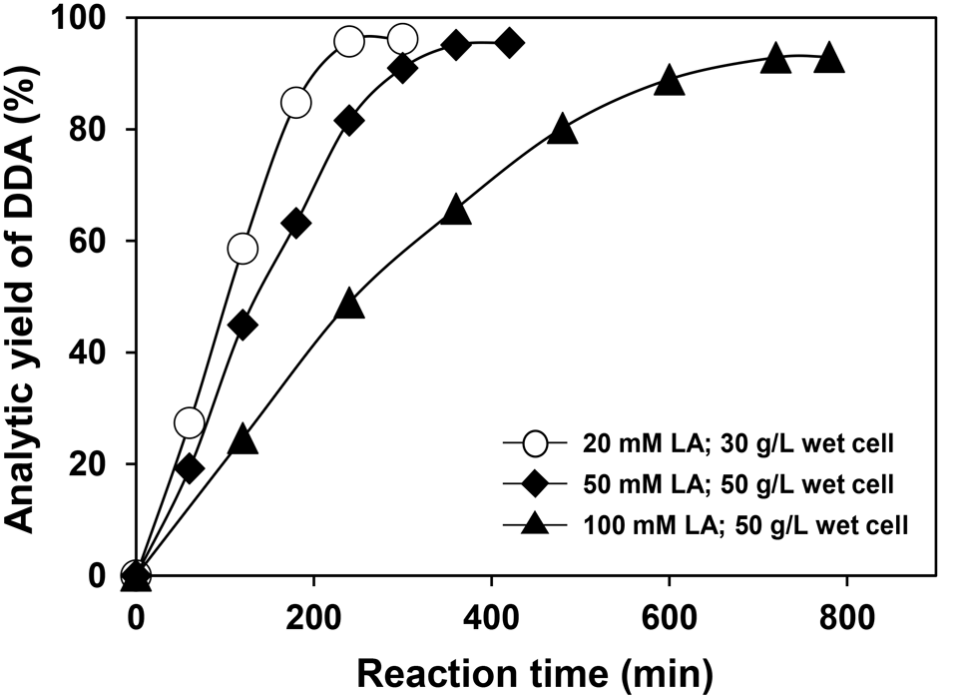
Reaction time courses for the conversion of linoleic acid to DDA by wet cells of *E. coli* BL21-DE3 harboring p-HRGS-2 with different substrate loadings (20, 50, and 100 mM)

The *E. coli* BL21(DE3) cells harboring p-HRGS-2 were employed as the whole-cell catalyst for the multi-enzymatic cascade conversion of LA. The whole-cell one-pot synthesis of DDA was performed at different LA loadings and cell dosages (Fig. 5). With a cell dosage of 30 g L^–1^, 4.41 g L^–1^ of DDA was produced from 20 mM of LA after 4 h of reaction, giving a molar yield of 95.7% and a productivity (STY) of 26.5 g L^− 1^ d^− 1^. Then, LA loading was raised to 50 mM with an increased cell dosage of 50 g L^–1^, and a molar yield of 95.1% was achieved after 6 h of reaction, giving an STY of 43.8 g L^− 1^ d^− 1^. When the LA loading was further increased to 100 mM, the highest molar yield of 92.9% was obtained after 12 h of reaction, and the STY of DDA dropped slightly to 42.8 g L^− 1^ d^− 1^. In addition, when the LA loading was 50 mM, the increased cell dosage of higher than 50 g L^–1^ led to a decreased STY, which might be attributed to the poor mass transfer efficiency in the reaction system caused by the increase of cell density. It is worth noting that there was a 5 ∼ 7% difference between the analytical yield and the theoretical yield of the product, which was likely to be caused by the consumption of LA or the intermediates by other metabolic reactions in the cells.

The product was isolated from the reaction mixture, purified by silica gel column chromatography, and characterized by ^1^H-NMR and ^13^C-NMR (Additional file 1: Fig. S5).

## Conclusions

In summary, by recruiting 13-lipoxygenase, 13-hydroperoxide lyase, aldehyde dehydrogenase, double bond reductase and NADH oxidase, a sustainable and eco-friendly DDA biosynthesis route was established by further development of the lipoxygenase pathway. After optimization of the rate-limiting step in the cascade route, whole-cell one-pot synthesis of DDA from renewable linoleic acid was performed with a high productivity of 43.8 g L^− 1^ d^− 1^, which is the highest record among the current LOX-pathway derived multi-enzymatic routes, and is the second highest record among the current research progress of DDA biosynthesis (Table 1). This work provides a new feasible route for DDA bioproduction, which may shed light on practical applications of industrial manufacture. Moreover, our work further expands the utilization of renewable linoleic acid resource, accelerating the development of sustainable industry and society.

**Table 1:** Overview of studies on the biosynthesis of dodecanedioic acid

Substrate	Strain	DDA titer (g/L)	STY (g·L^− 1^·d^− 1^)	Ref.
*n*-Dodecane	*C. tropicalis* S 76	2.1	1.4	Yi et al. 1982
Dodecanoic acid	*C. cloacae* FERM P736	5	1.2	Green et al. 2000
*n*-Dodecane	*C. tropicalis* CGMCC 356	166	33.1	Liu et al. 2004
*n*-Dodecane	*C. tropicalis* ATCC 20962	17	12.0	Mishra et al. 2016
*n*-Dodecane	*C. viswanathii* ipe-1	181.6	38.2	Cao et al. 2017b
Dodecanoic acid methyl ester	*C. tropicalis* ATCC 20962	66	8.4	Funk et al. 2017
*n*-Dodecane	*C. viswanathii* ipe-1	201.3	NA	Cao et al. 2018
Dodecanoic acid methyl ester	*W. sorbophila* UHP4	92.5	19.9	Lee et al. 2018
Dodecanoic acid	*S. cerevisiae* BCM	3.8 × 10^− 5^	7.6 × 10^− 6^	Buathong et al. 2020
12-Hydroxy dodecanoic acid	recombinant *E. coli*	6.9	13.8	Lim et al. 2021
Dodecanoic acid	recombinant *E. coli*	0.53	NA	Lim et al. 2021
*n*-Dodecane	*C. viswanathii*	224	44.9	Pham et al. 2023
Linoleic acid	recombinant *E. coli*	10.95	43.8	This work

### Electronic supplementary material

Below is the link to the electronic supplementary material.

Supplementary Material 1

## Data Availability

All data generated or analyzed during this study are included in this article and its Additional files.

## Abbreviations

*Ri*-LOX: lipoxygenase from *Rivularia* sp. PCC 7116
*Hv*-HPL: hydroperoxide lyase from *Hordeum vulgare*
*Gt*-ALDH: aldehyde dehydrogenase from *Geobacillus thermodenitrificans*
*St*-NOX: NADH oxidase from *Streptococcus thermophilus*
MBP: maltose-binding protein
LA: linoleic acid
13-HPOD: 13-hydroperoxy-9,11-(*Z*,*E*)-octadecadienoic acid
DDA: dodecanedioic acid
CYP: cytochrome P450 monooxygenase
