# Supplementary material for: Whole-cell one-pot biosynthesis of dodecanedioic acid from renewable linoleic acid
Source: Bioresour Bioprocess. 2024 May 23;11(1):55. doi: 10.1186/s40643-024-00770-8 (PMC11116355; doi:10.1186/s40643-024-00770-8)
Supplement: Supplementary file 1 — Supplementary Material 1 [file 40643_2024_770_MOESM1_ESM.docx]

Supporting Information for

**Whole-Cell One-Pot Biosynthesis of Dodecanedioic Acid from Renewable Linoleic Acid**

Yi-Ke Qi^1,2^, Jiang Pan^1^, Zhi-Jun Zhang^1^* and Jian-He Xu^1^*

^1^ State Key Laboratory of Bioreactor Engineering, School of Biotechnology, East China University of Science and Technology, 130 Meilong Road, Shanghai, 200237, China

^2^ College of Food Science and Biology, Hebei University of Science and Technology, 26 Yuxiang Street, Shijiazhuang, 050018, China

*Corresponding authors. E-mails: zjzhang@ecust.edu.cn; jianhexu@ecust.edu.cn.

**Table S1** Primers used for the construction of co-expression system.

| Primers | 5’ to 3’ Sequence |
| --- | --- |
| LOX-F | CCGCAGCCGCAACCGAGCAGCTAATTTGTTTAACTTTAAGAAGGAGA |
| LOX-R | GTAAAACGACGGCCAGTGCCAAGCTTTTAAATGTTGATGCTCTG |
| ALDH-F | CGCAGAGCATCAACATTTAATTTGTTTAACTTTAAGAAGGAGATATACCATGATCTACGCTCAACCGGGTC |
| ALDH-R | GTAAAACGACGGCCAGTGCCAAGCTTTTAGAACAGACCCAGCTTTT |
| NOX-F | CCCGAAAAAGCTGGGTCTGTTCTAATTTGTTTAACTTTAAGAAGGAGATATACCATGTCAAAAATCGTAGTTG |
| NOX-R | GTAAAACGACGGCCAGTGCCAAGCTTTTATTCAGCGGAGATAGCTG |
| 3-F | CTTTAAGAAGGAGATATACCATGATCTACGCTCAACCGGG |
| 3-R | CAACTACGATTTTTGACATGGTATATCTCCTTCTTAAAGTTAAACAAATTAGAACAGACCCAGCTTTTTCGGG |
| 4(NOX)-F | CCCGAAAAAGCTGGGTCTGTTCTAATTTGTTTAACTTTAAGAAGGAGATATACCATGTCAAAAATCGTAGTTG |
| 4-R | GTGGTGCTCGAGTGCGGCCGCTTATTCAGCGGAGATAGCTGC |
| Pci-F | GCCTTTTGCTGGCCTTTTGCTCCAAAAAACCCCTCAAGACCCG |
| Pci-R | GGGGATAACGCAGGAAAGAACATGTTAATACGACTCACTATAGGGG |

**Scheme S1**. Synthesis of green leaf volatile (GLVs) compounds from linoleic acid following the LOX pathway in plants (ADH: alcohol dehydrogenase).

**Figure S1.** SDS-PAGE analysis of the heterologous expression of *Ri*-LOX (A), *Gt*-ALDH and *St*-NOX (B). A: The heterologous expression of *Ri*-LOX by vector pET-21a in *E. coli* BL21(DE3) (lane 1: cell free lysate supernatant of *Ri*-LOX; lane 2: precipitate of cell free lysate); B: The heterologous expression of *Gt*-ALDH and *St*-NOX by vector pET-28a in *E. coli* BL21(DE3), respectively (lane 1: cell free lysate supernatant of *Gt*-ALDH; lane 2: cell free lysate supernatant of *St*-NOX).

**Figure S2.** SDS-PAGE analysis of the heterologous expression of *Hv*-HPL (53.4 kDa). A: The heterologous expression of *Hv*-HPL by vector pET-21a in *E. coli* BL21(DE3) with different IPTG concentrations (0.05~0.2 mM) and induction temperatures (16 ℃ or 28 ℃); B: The heterologous expression of *Hv*-HPL by vector pQE-30 in *E. coli* BL21(DE3) with different IPTG concentrations (0.2~1 mM); C: The heterologous expression of *Hv*-HPL by vector pMal-c2X in *E. coli* JM-109 at different temperatures (16 ℃ or 28 ℃). (P: precipitate; S: supernatant)


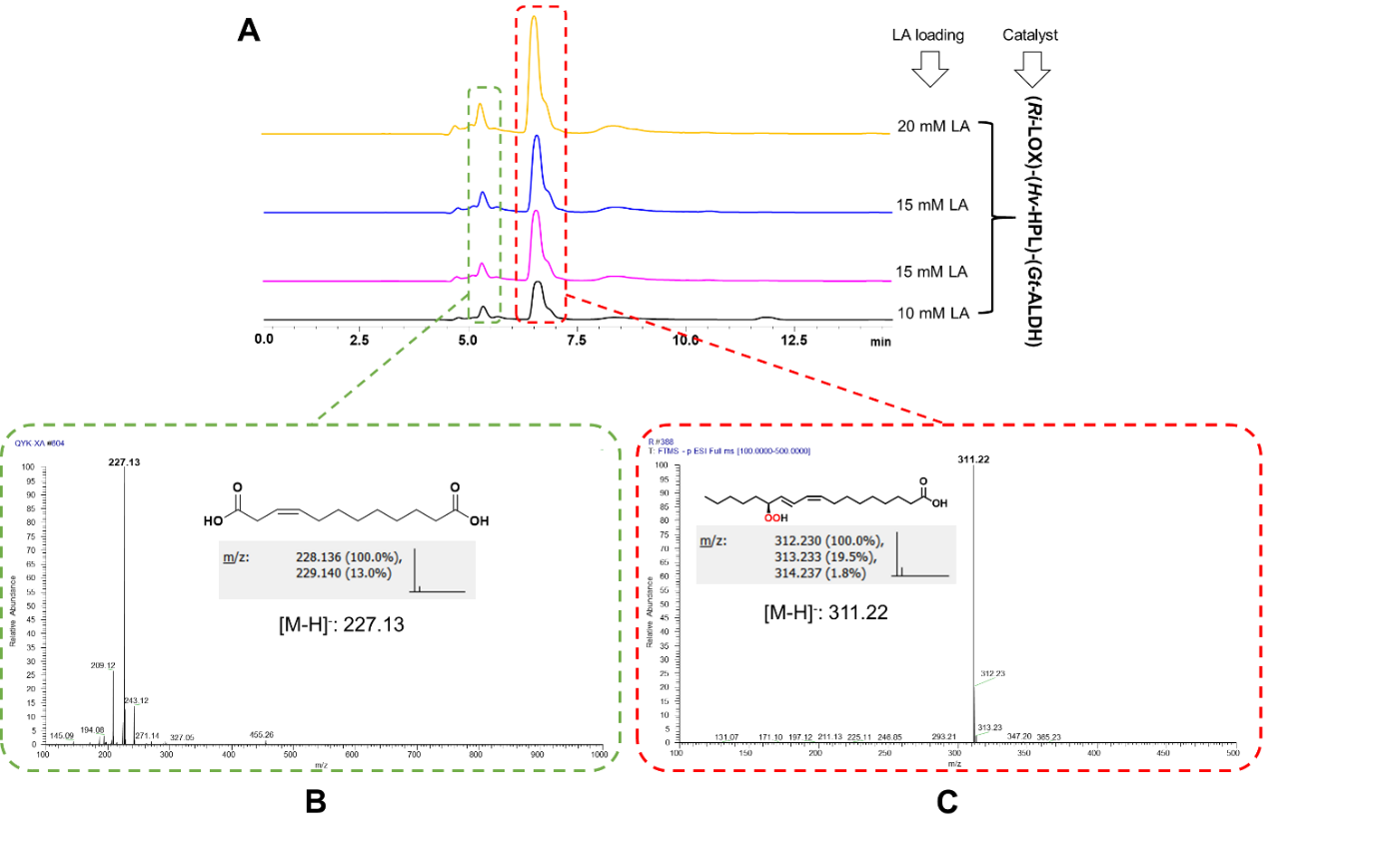


**Figure S3**. LC-MS analysis of the three-step cascade reaction. A. Liquid chromatogram of the reaction extract; B. High resolution mass spectra (anion) of 3*Z*-DEDA; C. High resolution mass spectra (anion) of 13*S*-HPOD.

**Figure S4**. SDS-PAGE analysis of the protein expression of two co-expression systems (Lane 1, 2: p-HRGS-1 in *E. coli* JM-109; Lane 3, 4: p-HRGS-1 in *E. coli* BL21-DE3; Lane 5, 6: p-HRGS-2 in *E. coli* BL21-DE3)

**Figure S5**. ^1^H-NMR (**a**) and ^13^C-NMR (**b**) spectra of the isolated product, DDA.

**Figure S6**. HPLC analysis of the product dodecanedioic acid produced from linoleic acid by multi-enzymatic whole-cell catalysis, using the elution buffer containing methanol/water/phosphoric acid (95:5:0.1) at a flow rate of 0.4 ml min^−1^ and at a detection wavelength of 210 nm.
